# The Curious Case of Lennox-Gastaut Syndrome: Treatment-Resistant Seizures in a Patient With Autism Spectrum Disease With Lennox-Gastaut Syndrome

**DOI:** 10.7759/cureus.16784

**Published:** 2021-07-31

**Authors:** Priyanka Anvekar, Petras Lohana, Mohammed Elmahal, Syed R Ali

**Affiliations:** 1 Internal Medicine, Mahatma Gandhi Mission (MGM) Medical College and Hospital, Mumbai, IND; 2 Internal Medicine, Liaquat University of Medical and Health Sciences Hospital, Karachi, PAK; 3 Internal Medicine, University of London, London, GBR; 4 Internal Medicine, Civil Hospital, Dow University of Health Sciences, Karachi, PAK

**Keywords:** autism spectrum disease, epileptic seizures, anti-epileptic drugs, mental retardation, lennox-gastaut syndrome

## Abstract

Lennox-Gastaut syndrome (LGS) is a childhood epilepsy disorder seen between the ages of one to eight years with the electroencephalogram (EEG) changes showing slow spiked-wave complex bursts or paroxysms of generalized fast activity and intellectual disability and often needing multiple lines of treatment. Autism spectrum disease (ASD) is rare but catastrophic comorbidity seen in a patient with LGS. We report an eight-year-old boy presenting to the emergency department with seizures and mental retardation. His first seizure was at the age of five months but was symptomatically treated without any specific diagnosis. On further investigation, the patient was diagnosed with LGS with concomitant ASD. The patient has successfully been treated for his treatment-resistant seizures and is now on regular follow-ups. This article aims to highlight this rare combination of LGS along with ASD and understand the disease course.

## Introduction

Lennox-Gastaut syndrome (LGS) is a severe intractable seizure disorder of childhood-onset [1]. Age of onset is usually one to eight years and presents with the characteristic triad of multiple drug-resistant seizures, electroencephalogram (EEG) changes, and associated intellectual disability [1]. The management options may vary from anti-epileptic drugs and non-pharmacological options from the ketogenic diet to vagus nerve stimulation and corpus callosotomy [1]. Autism spectrum disease (ASD) is an uncommon childhood disorder with a prevalence of 3% in children [2]. It has been observed that children with ASD often show the manifestation of seizures and this is thought to be the cognitive impairment in many patients [2]. There have been a few cases reported of LGS with ASD in the past but this remains a rare entity [3]. We report a case of a young boy presenting with treatment-resistant seizures and diagnosed with LGS and ASD. His treatment, prognosis, and outcome have been addressed and discussed further in this report.

## Case presentation

**Figure 1:**
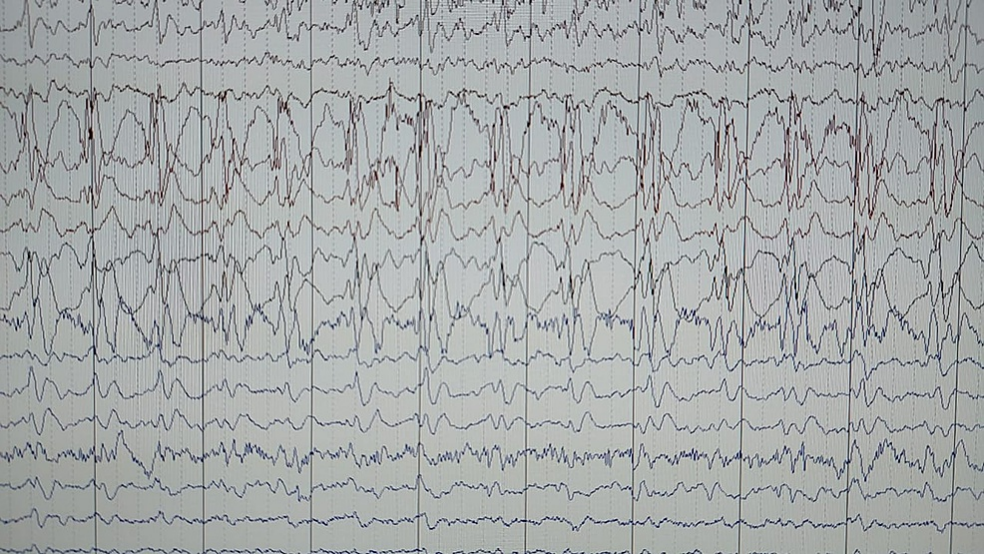
EEG demonstrating fast activity and slow-wave discharge.

An eight-year-old boy presented to the emergency department with complaints of generalized tonic conic seizures (GTCS). He had one episode of GTCS about 20 minutes before the arrival, which lasted for three minutes followed by loss of consciousness. The patient was born at 38 weeks of gestation via normal delivery and had an uneventful course of birth. He has a family history of seizures in his father and paternal uncle controlled on medications. He had his first GTCS at the age of five months after a febrile episode which was recurrent for almost 30 minutes duration. However, his parents never followed him up for medical care due to financial constraints, and therefore was never diagnosed with any specific condition. A neurology consult was given. His EEG in the hospital showed changes supporting LGS (Figure 1).

Brain magnetic resonance imaging (MRI) showed nothing of significance. The patient has a history of talipes equinovarus correction done at the age of seven months. He has no history of perinatal distress, infection, encephalitis, exanthems. He was noted to be resistant to various anti-epileptic drugs tried in the past, such as carbamazepine 75 mg two times per day and phenytoin 50 mg three times per day, and has reported many episodes of GTCS. On examination, the patient was hyperactive and difficult to control but had no harmful tendencies. He was noted to repetitively arrange the items from his mother’s purse in a particular order. He did not make any eye contact with anyone in particular. His vitals were normal. The complete blood count, basic metabolic panel, liver, and renal panel were within normal limits. The psychiatry team was consulted. The patient was assessed for ASD with Autism Diagnostic Observation Schedule (ADOS) and diagnosis of autism spectrum disease (ASD) was eventually established. The patient’s medications were revised and the patient was started on piracetam 400 mg in the morning and 800 mg in the evening, sodium valproate 200 mg every six-hour, and clonazepam 0.5 mg in the morning and 1.5 mg in the evening and was given knowledge about adjuvant therapy like occupational and behavioral therapy. He was sent home and advised of regular follow-ups. The patient’s parents were thoroughly counseled on the importance of follow-up and medication compliance. His follow-up visits reported great control of seizures. However, his ASD continued to show no improvement. He is continued on his anti-epileptic medications and follows up regularly with his speech and occupational therapist.

## Discussion

Lennox-Gastaut syndrome is a severe form of neurodegenerative disorder affecting children mainly between the age of one to eight years but can affect other age groups as well. The diagnosis is established by the triad of drug-resistant seizures, EEG with slow spike and wave pattern, and cognitive impairment. However, few patients may not present with the triad at once and vary in the onset of symptoms [4]. "Drop attacks" comprise multiple variants of seizures such as GTCS or atonic followed by a fall [4]. Although many types of seizures can be seen in LGS, generalized paroxysmal fast activities (GPFA) and tonic-clonic seizures and atonic are most commonly observed and support the diagnosis of LGS. The course of LGS may change over time as the patients reach adulthood and the severity of seizures may decline. However, GTCS may persist over time and may be more pronounced during sleep [5]. The incidence of LGS is very rare and is seen in approximately 0.2-2.8/10,000 births annually with predominance for the male gender. Around 70-75% of the patients diagnosed with LGS have shown underlying atypical brain features. These may include various pathologies such as developmental malformations, neurocutaneous syndromes, post-hypoxic-ischemic insult, meningitis, encephalitis, or metabolic encephalopathy [6]. The exact pathophysiology remains unknown. Thirty percent of patients had a family history of epilepsy [7]. Patients with SCN1A gene mutation may not exhibit the characteristic features of LGS. It was hypothesized that the SCN1A splicing was the cause of the myoclonic type of seizures [8]. Another study has shown an association between LGS and human lymphocyte antigen (HLA) class1 antigen B7 [9]. The EEG changes typical of LGS may not be appreciated right after the seizure [9]. In our patient, a follow-up EEG showed the typical features. The typical finding on the EEG is a slow spike-wave of <3 Hz with an atypical background. LGS has been noted to involve the frontal lobe in many of the documented cases. The slow spike-wave mainly appears in the frontal lobe and thus we can anticipate the neurologic impairment in patients with LGS [9]. The characteristic slow epileptiform discharges are generalized and bilaterally synchronous occurring simultaneously in bursts of varying duration. Generalized paroxysmal fast rhythms are experienced during sleep and suggest nocturnal tonic-clonic seizures [4,10]. Radiologic investigations such as MRI are preferred to further analyze the disease whereas a computed tomography (CT) scan is advised in case of any head injury due to seizures. Autism Spectrum Disease(ASD) is the developmental disorder of the brain requiring the presence of a continuous deficit in social communication and interaction and the presence of stereotyped patterns of behavior, interests, and activities [11]. ADS is known to be measured most conveniently by Autism Diagnostic Observation Schedule (ADOS), although there are many other scales available for diagnosing ADS as well [11]. One of the risk factors for ASD in LGS was thought to be seizures. Early-onset of seizures before the age of two years mainly leads to neurodegeneration and ASD. However, it was noted that the co-morbidity of ASD in LGS was comparatively lower than the other epileptic diseases such as Dravet syndrome [12]. Our patient showed the typical finding of ASD in conjunction with LSD. The aim of treatment in patients with LGS is mainly to achieve seizure control and improve the life quality in these patients. There are various pharmacological and non-pharmacological modalities proposed for the treatment of LGS. The commonly used anti-epileptic drugs are sodium valproate, lamotrigine, benzodiazepines, phenytoin sodium which can be used in combination if the seizures are not controlled with monotherapy with either of the drugs [13-15]. Other non-pharmacological treatment options which have proven to be highly beneficial in a patient with LGS are the ketogenic diet, vagus nerve stimulation, and surgical intervention. The ketogenic diet comprises of high fat and low carbohydrate diet and has shown fruitful results in patients with LGS in regards to seizure control. Vagal nerve stimulation can be helpful in seizure control but the improvement noted in seizures is gradual and may require increased stimulation intensity over time [16]. The mechanism of action is unclear however this technique helps with mood and cognition and therefore helps with intellectual disability [17]. Surgical interventions like partial or complete callosotomy is a palliative treatment option that has shown drastic improvement in the drop attacks. The improvement could be dramatic post-surgery but the recurrence can persist [18]. Our patient presented with treatment-resistant seizures but finally achieved control with a change in the line of treatment.

Lennox-Gastaut syndrome generally has a poor outcome. Almost 80% of patients will have drug-resistant epilepsy and tonic-clonic seizures in sleep. There has been some evidence that newer AEDs if incorporated early can help limit cognitive impairment, however, seizure control is a must. Over 50% of mortalities are seen to be due to seizure-related complications [19]. Sudden expected death in epilepsy (SUDEP) has also been documented in few studies to be the reason for death in children with LGS [20]. Lastly, the long-term prognosis of children with LGS differs greatly but it's very rare to get completely rid of the seizures. Most children show permanent cognitive impairment and demonstrate ongoing behavioral problems. The patients experience seizures even in adulthood, with the type and frequency changing over time and should be addressed with regular medical care. Patients with LGS have an increased risk of premature mortality, often from a sudden unexpected death in epilepsy or due to any underlying brain disorder.

## Conclusions

In summary, ASD is a rare but devastating co-morbidity associated with Lennox-Gastaut syndrome. There are many possible etiologies for cognitive impairment but seizures are known to be the root cause for the development of ASD in children with Lennox-Gastaut syndrome. The prognosis of these children remains poor and management mainly comprises seizure management by medical, non-medical, or surgical means and occupational, behavioral, and educational therapy to support the patients with co-existing ASD. Family counseling plays a vital role and should be incorporated at a very early stage while managing the patients.
